# Whole genome sequencing of drug resistant *Mycobacterium tuberculosis* isolates from a high burden tuberculosis region of North West Pakistan

**DOI:** 10.1038/s41598-019-51562-6

**Published:** 2019-10-18

**Authors:** Abdul Jabbar, Jody E. Phelan, Paola Florez de Sessions, Taj Ali Khan, Hazir Rahman, Sadiq Noor Khan, Daire M. Cantillon, Leticia Muraro Wildner, Sajid Ali, Susana Campino, Simon J. Waddell, Taane G. Clark

**Affiliations:** 1 Department of Medical Lab Technology, University of Haripur, Haripur, Pakistan; 2 Department of Microbiology, Kohat University of Science and Technology, Kohat, Pakistan; 3 Faculty of Infectious and Tropical Diseases, London School of Hygiene and Tropical Medicine, Keppel Street, London, WC1E 7HT UK; 4 Genome Institute of Singapore, 60 Biopolis St, Biopolis, Singapore; 5 Department of Microbiology, Abdul Wali Khan University, Mardan, Pakistan; 6 Department of Global Health and Infection, Brighton and Sussex Medical School, University of Sussex, Brighton, BN1 9PX UK; 7 Provincial Tuberculosis Reference Laboratory, Hayatabad Medical Complex Peshawar, Khyber Pakhtunkhwa, Pakistan; 8 Faculty of Epidemiology and Population Health, London School of Hygiene and Tropical Medicine, Keppel Street, London, WC1E 7HT UK

**Keywords:** Microbial genetics, Genome evolution

## Abstract

Tuberculosis (TB), caused by *Mycobacterium tuberculosis* bacteria, is a leading infectious cause of mortality worldwide, including in Pakistan. Drug resistant *M. tuberculosis* is an emerging threat for TB control, making it important to detect the underlying genetic mutations, and thereby inform treatment decision making and prevent transmission. Whole genome sequencing has emerged as the new diagnostic to reliably predict drug resistance within a clinically relevant time frame, and its deployment will have the greatest impact on TB control in highly endemic regions. To evaluate the mutations leading to drug resistance and to assess for evidence of the transmission of resistant strains, 81 *M. tuberculosis* samples from Khyber Pakhtunkhwa province (North West Pakistan) were subjected to whole genome sequencing and standard drug susceptibility testing for eleven anti-TB drugs. We found the majority of *M. tuberculosis* isolates were the CAS/Delhi strain-type (lineage 3; n = 57; 70.4%) and multi-drug resistant (MDR; n = 62; 76.5%). The most frequent resistance mutations were observed in the *katG* and *rpoB* genes, conferring resistance to isoniazid and rifampicin respectively. Mutations were also observed in genes conferring resistance to other first and second-line drugs, including in *pncA* (pyrazinamide), *embB* (ethambutol), *gyrA* (fluoroquinolones), *rrs* (aminoglycosides), *rpsL, rrs* and *giB* (streptomycin) loci. Whilst the majority of mutations have been reported in global datasets, we describe unreported putative resistance markers in *katG*, *ethA* (ethionamide), *gyrA* and *gyrB* (fluoroquinolones), and *pncA*. Analysis of the mutations revealed that acquisition of rifampicin resistance often preceded isoniazid in our isolates. We also observed a high proportion (17.6%) of pre-MDR isolates with fluoroquinolone resistance markers, potentially due to unregulated anti-TB drug use. Our isolates were compared to previously sequenced strains from Pakistan in a combined phylogenetic tree analysis. The presence of lineage 2 was only observed in our isolates. Using a cut-off of less than ten genome-wide mutation differences between isolates, a transmission analysis revealed 18 *M. tuberculosis* isolates clustering within eight networks, thereby providing evidence of drug-resistant TB transmission in the Khyber Pakhtunkhwa province. Overall, we have demonstrated that drug-resistant TB isolates are circulating and transmitted in North West Pakistan. Further, we have shown the usefulness of whole genome sequencing as a diagnostic tool for characterizing *M. tuberculosis* isolates, which will assist future epidemiological studies and disease control activities in Pakistan.

## Introduction

Tuberculosis (TB), caused by *Mycobacterium tuberculosis* bacteria, is a global public health problem responsible for 10 million new cases and 1.6 million deaths worldwide in 2017^1^. *M. tuberculosis* drug resistance is making disease control more difficult, with 490,000 TB cases identified as resistant to both rifampicin (RIF) and isoniazid (INH) (multi-drug resistant, “MDR-TB”) in 2017. Five countries India, China, Indonesia, Philippines and Pakistan contribute more than half (56%) of the total TB global burden^1^. Pakistan has an estimated 518,000 TB cases each year, including ~15,000 MDR-TB patients. The estimated proportion of MDR-TB in Pakistan is ~4% in new cases and ~17% in patients who have previously been treated^2^. Khyber Pakhtunkhwa province (population size 35.5 million; 11.9% of the total population) is situated in North West Pakistan and shares a border with Afghanistan. The province contains the semi-autonomous federally administered tribal areas inhabited by the Pashtun people. The province has been affected by recent military action and accommodates the majority of the 1.4 million Afghan refugees currently in Pakistan. Khyber Pakhtunkhwa has an estimated 270 TB cases per 100,000 population^2^. Sputum smear microscopy is used as a primary screening test for the diagnosis of TB at local clinics, while GeneXpert MTB/RIF assays are employed for the rapid detection of rifampicin resistant TB at the district level^3^. Laboratory culture and drug susceptibility testing are available at the provincial level. Treatment of drug-susceptible TB is for six months, while for MDR-TB it is nearly two years^4^. *M. tuberculosis* may become extensively drug resistant (XDR-TB), which is MDR-TB with additional resistance to fluoroquinolones (e.g. ofloxacin) and at least one of the second line injectable aminoglycoside drugs (e.g. kanamycin, amikacin or capreomycin)^5^. In Pakistan, of all TB cases, ~5% are MDR-TB, and of these, ~5% are XDR-TB^6,7^.

The global emergence and rise in the prevalence of MDR-TB and XDR-TB cases in the past decade has made it imperative to detect drug resistance rapidly and accurately^8^. Drug resistance in *M. tuberculosis* is almost exclusively due to mutations (including single nucleotide polymorphisms (SNPs), insertions and deletions (indels)) in genes coding for drug-targets or drug-converting enzymes^9,10^. Putative compensatory mechanisms have been described to overcome fitness impairment that arise during the accumulation of resistance conferring mutations^9,11^. Efflux pumps are also thought to have a role in resistance^9,12^. *M. tuberculosis* culture and drug susceptibility testing is the gold standard technique, but this can take several weeks. The development of molecular tests, such as GeneXpert and line probe assays, can be used to detect *M. tuberculosis* directly from clinical samples and identify some mutations underlying MDR-TB. Whole genome sequencing (WGS) provides higher resolution^9^, and can be used to identify SNPs and indels in loci linked to drug resistance^13^. Known and putative markers of drug resistance have been identified using phylogenetic tree-based and genome-wide association study approaches^9^. Libraries of informative resistance mutations are leading to the development of informatic tools to rapidly profile samples for their drug susceptibility to aid clinical decision making^10,14^.

The *M. tuberculosis* complex has seven lineages that are endemic in different locations around the globe, leading to the hypothesis that the strain-types are specifically adapted to people of different genetic backgrounds^15,16^. The lineages vary in their geographic distribution and spread, with lineage 2 being particularly mobile with evidence of recent spread from Asia to Europe and Africa^15^. Lineage 4 is common in Europe and southern Africa, with regions of high TB incidence and high levels of HIV co-infection. Lineage 3, including Central Asian (CAS) strains, are common in South Asia. The lineages may vary in propensity to transmit and severity of disease^16^, but there is considerable inter-strain variation within lineages^12^. A set of SNPs has been identified that can be used to barcode sub-lineages^15^, leading to informatic tools that position sequenced samples within a global phylogeny^17^. Similarly, SNPs have been used to construct transmission networks, where samples from different individuals that have near identical genome sequences are most likely to be due to a transmission event. Analysis of genome-wide SNPs characterized in *M. tuberculosis* DNA sourced from a highly endemic TB region in Malawi has shown striking differences by lineage in the proportion of disease due to recent transmission and in transmissibility, highest in lineage2 (East-Asian), and lowest in lineage1 (Indo-Oceanic)^18^.

There have been few WGS TB studies in Pakistan, and none have focused on the tribal and migrant populations of the North West. One study characterized drug resistance mutations across 42 XDR-TB isolates from the Aga Khan University (Karachi) strain bank (years 2004–2009), which were sourced from 4 provinces (Sindh (21), Punjab (16), Khyber Pakhtunkhwa (4), Baluchistan (1))^19^. These isolates were predominantly CAS lineage 3 strains^19^, in keeping with previous genotyping-based studies^20^. The Karachi study found that most rifampicin resistance was attributable to SNPs in the *rpoB* hot-spot region, and isoniazid resistance was most commonly associated with the *katG* (codon 315) and *inhA (*S94A) mutations. Beyond MDR-TB, the study found that only 43% of pyrazinamide could be explained by *pncA* SNPs, fluoroquinolone resistance was mostly explained by *gyrA* (91–94 codon) mutations, and resistance to aminoglycoside injectables was associated with *rrs* mutations. The concordance between phenotypic and genotypic testing was highest for rifampicin and isoniazid (>90%), and lowest for pyrazimamide (<50%)^19^. Follow-up work with the XDR-TB isolates revealed SNPs in efflux pump genes, which may influence drug resistance^12^. In our study, we performed WGS on 81 drug resistant *M. tuberculosis* from the Khyber Pakhtunkhwa province, which is endemic for TB across its tribal and migrant populations, but where public health surveillance systems are not strong. We characterize the underlying *M. tuberculosis* resistance mutations, identifying novel drug-resistance conferring mutations and, in a combined Pakistan WGS data analysis, reveal potential MDR-TB transmission chains. Our methods and findings will assist future WGS and drug resistance mapping studies and inform disease control efforts in Pakistan and neighbouring Afghanistan.

## Materials and Methods

### The samples and whole genome sequencing

A total of 81 predominantly drug resistant isolates were randomly selected from 8,220 archived *M. tuberculosis* samples collected between June 2016 and June 2017 at the Provincial TB Reference Laboratory, Hayatabad Medical Complex Peshawar, Khyber Pakhtunkhwa province of Pakistan. Demographic data (e.g. age, sex) were collected from each TB patient that contributed sputum, alongside drug regimen and treatment outcome. The sputum samples were digested and decontaminated using the N-acetyl-L-cysteine sodium hydroxide (NALC-NaOH) method, and the *M. tuberculosis* cultured in modified 7H9 Middlebrook media in the Mycobacterium Growth Indicator Tube (MGIT) system. Positive cultures were confirmed using the BD MGIT TBc identification (TBc ID) or Capilia chromatographic tests^21^. All laboratory work involving the culture of live bacteria (from sputum) was performed under category level 3 bio-containment facilities and protocols. DNA samples were extracted using the CTAB method^22^. Before sequencing, the DNA was RNase-treated, quantified and quality assessed by NanoDrop One spectrophotometer and Qubit 2.0 fluorometer using the Qubit dsDNA BR Assay Kit (ThermoFisher Scientific). The samples were sequenced on the Illumina MiSeq and HiSeq2000 platforms using 200 bp paired end runs at the London School of Hygiene and Tropical Medicine and Genome Institute of Singapore genomic facilities. The confirmed *M. tuberculosis* MGIT cultured isolates underwent standard drug susceptibility testing against isoniazid (critical concentration 0.1 µl/ml), rifampicin (1.0 µl/ml), ethambutol (5.0 µl/ml), streptomycin (1.0 µl/ml), moxifloxacin (2.5 μg/ml), amikacin (1.0 μg/ml), kanamycin (2.5 μg/ml), capreomycin (2.5 μg/ml) and ofloxacin (2.0 μg/ml). Pyrazinamide susceptibility testing was performed using an established protocol^23^.

### Bioinformatic analysis

Sequence reads were inspected using fastQC (www.bioinformatics.babraham.ac.uk/projects/fastqc/) as a primary assessment of data quality. The reads were trimmed using trimmomatic software^24^ to remove low quality sequences, and then mapped against the H37Rv reference genome (AL123456) using the BWA-mem alignment package^25^. SNPs were called using the BCF/VCF software suite^26^, and those in non-unique regions of the genome (e.g. *ppe* genes) were excluded. SNPs were converted into a FASTA format alignment, which was used as input to RAxML (v8.0.0) software^27^ to reconstruct a phylogenetic tree. The tree was annotated and visualized using iTOL^28^. Drug resistance profiles and lineages were predicted *in-silico* using TB-Profiler (v2.4)^10,14^, using a library of established mutations (https://github.com/jodyphelan/tbdb). SpolPred software^29^ was used to *in-silico* predict spoligotypes.

### Ethical approval

This study was approved by the ethical committees of the Kohat University of Science and Technology, Kohat, and the Provincial TB Reference Lab, Hayatabad Medical Complex Peshawar, Pakistan. Informed consent was given by all patients who contributed sputum.

## Results

### Clinical isolates and phylogeny

**Figure 1:**
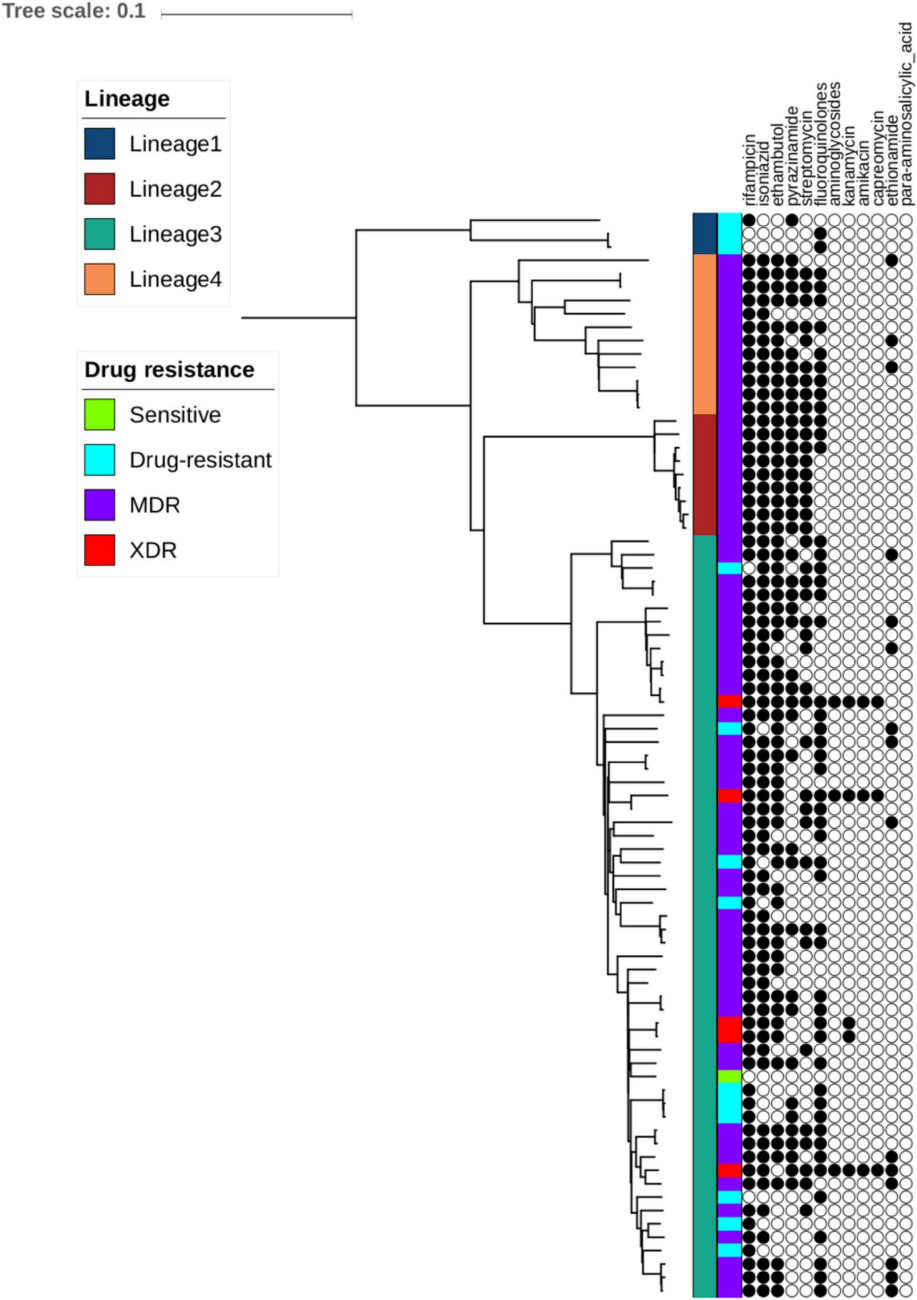
Phylogenetic tree of *M. tuberculosis* strains (n = 81), with their lineages and drug resistance profiles. The phylogenetic tree was created using a maximum likelihood approach implemented in RAxML^27^. The tree was annotated using iTOL^28^. The first vertical band to the right of the tree denotes the lineage. The second vertical band denotes the drug resistance phenotype. The circles show the drug resistance profiles, with filled circles representing the presence of a resistance mutation. Some profiles are examples of isolates that are both pre-MDR and fluoroquinolone resistant. MDR = multi-drug resistant TB, XDR = extensively drug resistant TB; drug resistant = non-MDR/XDR resistant; based on drug susceptibility testing and TB-Profiler prediction^14^.

The 81 *M. tuberculosis* were predominantly sourced from TB hospitals and clinics in Peshawar, the largest city of Khyber Pakhtunkhwa province (Table 1**;** Supplementary Fig. 1). The patients contributing *M. tuberculosis* samples had a median age of 28, and there was gender parity (male: n = 41, 50.6%). *In-silico* predictions of resistance across the eleven anti-TB drugs revealed that the majority were MDR-TB (n = 62; 76.5%), with the others being pan-susceptible (n = 1; 1.2%), XDR-TB (n = 5; 6.2%) and non-MDR-/XDR-TB drug-resistant (n = 13; 16.0%). There were high levels of rifampicin (93.8%), isoniazid (84.0%), ethambutol (75.3%) and fluoroquinolone (63.0%) drug resistance and low levels of aminoglycoside resistance (3.7%). Mapping of the raw *M. tuberculosis* sequence data (Supplementary Table 1) led to high average genome-wide coverage across the clinical isolates (median: 227-fold; range: 74- to 288-fold). Across the isolates, 18,667 unique SNPs were identified; a high proportion (37%) of these were observed in single isolates. Isolates were classified predominantly into lineage 3 (CAS) strain types (70.4%) (Table 1), but lineages 1 (3.7%), 2 (11.1%; all Beijing strain-types) and 4 (14.8%) were also present. As expected, these lineages form clusters in a genome-wide SNP-based phylogenetic tree (Fig. 1**;** Supplementary Fig. 2).

**Table 1:** *Mycobacterium tuberculosis* samples (N = 81).

Characteristic	N	%
Male	41	50.6
Age (in years)		
5–14	4	4.9
15–24	24	29.6
25–54	40	49.4
>=55	13	16.1
Location*		
PMDT Peshawar	37	45.7
PMDT D.I.Khan	17	21.0
PMDT Abbottabad	13	16.0
PMDT Swat	6	7.4
PRL Peshawar	3	3.7
KTH Peshawar	2	2.5
HMC Peshawar	2	2.5
MMC Mardan	1	1.2
Lineages**		
1	3	3.7
2	9	11.1
3	57	70.4
4	12	14.8
Drug resistance***		
Rifampicin	76	93.8
Isoniazid	68	84.0
MDR-TB	67	82.7
Ethambutol	61	75.3
Streptomycin	40	49.4
Ethionamide	15	18.5
Fluoroquinolones	51	63.0
Aminoglycosides	3	3.7
XDR-TB	5	6.2

*HMC = Hayatabad Medical Complex, D.I.Khan = Dera Ismail Khan, KTH = Khyber Teaching Hospital, MMC = Mardan Medical Complex, PMDT = Programmatic Management of Drug Resistant TB, PRL = Provincial TB Reference Laboratory; **all lineage 3 strains are the CAS/Delhi strain-type; ***from *in-silico* prediction using TB-Profiler (v2.4)^14^, where MDR-TB = multi-drug resistant TB, XDR-TB = extensively drug resistant TB.

### Mutations underlying drug-resistance

Resistance mutations^14^ were assessed and compared to the phenotypic drug susceptibility test results for 11 anti-TB drugs. There was perfect concordance between the phenotypic result and *in-silico* prediction for rifampicin resistance. This resistance was predominantly associated with known mutations at codon 450 in the *rpoB* gene (conferred by 3 mutations; in 59/76 resistant isolates; including S450L 56/76; Table 2**;** Supplementary Table 2), but also ten other putative mutations in the rifampicin-resistance-determining region (RRDR) and two mutations outside this region (L430R, L430P). A minority of rifampicin resistant isolates had putative compensatory *rpoC* mutations (I491T 7/76 (all Beijing), I885V 1/76 (CAS/Delhi)), which all had *rpoB* S450L background mutations. Mutations identified in the *katG* gene (S315T 61/68) and the *Rv1482c*–*fabG1* intergenic region (7/68) were most likely to be responsible for isoniazid resistance, but additional mutations in *oxyR’-ahpC* (1/68) and *katG* (5/68) were found in single isolates (Table 2**;** Supplementary Table 2). The phenotypic results and *in-silico* predictions were identical except for three isolates. These isolates had distinct previously uncharacterized frameshift mutations in the *katG* gene (Supplementary Table 2**)**, most likely leading to resistance due to a loss of function of the isoniazid activating enzyme.

**Table 2:** High frequency drug resistance mutations. Mutations have been ordered by frequency.

Drug	Gene	Mutation	Resistant* (%)	Global frequency resistant (%)**
Isoniazid	*katG*	Ser315Thr	61/68 (89.7)	4071/5423 (75.1)
Rifampicin	*rpoB*	Ser450Leu	56/76 (73.7)	2975/4618 (64.4)
Fluoroquinolones	*gyrA*	Asp94Gly	26/51 (51.0)	153/527 (29.0)
Ethambutol	*embB*	Met306Ile	20/61 (32.8)	567/2662 (21.3)
Ethambutol	*embB*	Met306Val	15/61 (24.6)	840/2662 (31.6)
Streptomycin	*rpsL*	Lys43Arg	12/42 (28.6)	649/1408 (46.1)
Streptomycin	*rrs*	r.514a > c	12/42 (28.6)	128/1408 (9.1)
Ethambutol	*embB*	Met306Leu	10/61 (16.4)	34/2662 (1.3)
Fluoroquinolones	*gyrA*	Asp94Ala	9/51 (17.7)	62/527 (11.8)
Ethionamide	*fabG1*	−15C > T	7/15 (46.7)	190/347 (54.8)
Isoniazid	*fabG1*	−15C > T	7/68 (10.3)	877/5423 (16.2)
Rifampicin	*rpoC*	Ile491Thr	7/76 (9.2)	91/4618 (2.0)
Streptomycin	*gid*	c.102_102del	6/42 (14.3)	12/1408 (0.9)
Ethambutol	*embB*	Gln497Arg	5/61 (8.2)	207/2662 (7.8)
Fluoroquinolones	*gyrA*	Ser91Pro	5/51 (9.8)	40/527 (7.6)
Pyrazinamide	*pncA*	−11A > G	4/43 (9.3)	77/1946 (4.0)
Fluoroquinolones	*gyrA*	Ala90Val	4/51 (7.8)	152/527 (28.8)
Rifampicin	*rpoB*	Asp435Val	4/76 (5.3)	296/4618 (6.4)
Ethambutol	*embB*	Gly406Ala	4/61 (6.6)	318/2662 (12.0)
Pyrazinamide	*pncA*	Val180Phe	4/43 (9.3)	4/1946 (0.2)
Ethambutol	*embA*	−12C > T	3/61 (4.9)	115/2662 (4.3)
Fluoroquinolones	*gyrA*	Asp94Asn	3/51 (5.9)	22/527 (4.2)
Ethambutol	*embB*	Gln497Lys	3/61 (4.9)	20/2662 (0.8)
Ethambutol	*embB*	Gly406Asp	3/61 (4.9)	75/2662 (2.8)
Ethambutol	*embB*	Gly406Ser	3/61 (4.9)	37/2662 (1.4)
Rifampicin	*rpoB*	His445Tyr	3/76 (4.0)	230/4618 (5.0)
Pyrazinamide	*pncA*	His71Arg	3/43 (7.0)	2/1946 (0.1)
Streptomycin	*rpsL*	Lys88Arg	3/42 (7.1)	104/1408 (7.4)
Amikacin	*rrs*	1401a > g	3/3 (100)	291/349 (83.4)
Capreomycin	*rrs*	1401a > g	3/3 (100)	307/404 (76.0)
Kanamycin	*rrs*	1401a > g	3/5 (60.0)	531/669 (79.4)
Streptomycin	*rrs*	517c > t	3/42 (7.1)	57/1408 (4.1)

*The number of strains in this study predicted to be resistant by TB-Profiler software; **the number of strains in the global dataset (N > 18k)^14^ predicted to be resistant by TB-Profiler.

Ethambutol resistance was conferred by mutations in the *embCAB* operon, including *embB* (M306 45/61; G497 9/61; G406 7/61). By assuming the laboratory phenotypic test result as the gold standard, the sensitivity of the *in-silico* prediction was high (97.2%), but the specificity was much lower (42.2%). This differential is most likely due to the large number of isolates predicted to be resistant based on the presence of mutations in codon 306 of the *embB* gene (n = 10). These mutations have been shown to confer resistance, albeit at a moderate level^30^. Pyrazinamide resistance is typically associated with the *pncA* gene, and we identified 31 non-synonymous mutations in that locus, with the most frequent being *pncA-Rv2044c* −11A > G (4/43), V180P (4/43), and H71A (3/43). The comparison of the *pncA* allele frequencies in our study to those in a global WGS dataset^14^ revealed four indels and three SNPs to be novel (Supplementary Fig. 3, Supplementary Table 2**)**. Further, one isolate contained a 405 bp deletion that removed a large proportion of the *pncA* gene (Supplementary Fig. 3, Supplementary Table 2**)**. Mutations in other genes associated with pyrazinamide resistance (*rpsA* and *panD*) were not detected. There was a moderate level of concordance between the phenotypic drug susceptibility test results and *in-silico* predictions for pyrazinamide (sensitivity 74.1%, specificity 70.4%). However, this discrepancy may be explained by the known difficulty in performing drug susceptibility testing for pyrazinamide resistance and its resulting high variability.

As expected for the samples selected for sequencing, drug resistance conferring mutations were also detected to second line anti TB drugs. Streptomycin resistance is typically related to mutations in *gid, rpsL* and *rrs* loci, which are related to low, low and intermediate, and high levels of resistance, respectively^9^. The mutations occurring in the *gid* gene were a frameshift (102del, 6/42) and A80P (2/42). The most common resistance conferring mutations were in the *rrs* (514a > c; 12/42) and *rpsL* (L43A 12/42) genes. One of the isolates had mutations in both *gid* (G352 → GC) and *rpsL (*K88R). Resistance to ethionamide is associated with the *ethA* locus, and nine mutations were identified in that gene, each present in single resistant isolates (Supplementary Table 2). Eight of these mutations were not present in a large global resistance database^14^, and may be novel resistance conferring mutations. The most frequent mutation was in the *fabG1* promoter region (−15C > T; 7/15), which is also associated with isoniazid resistance^9^ (Table 2).

### Resistance to second-line fluoroquinolones and aminoglycosides

Mutations in the *gyrA* and *gyrB* loci associated with fluoroquinolone resistance were observed. The majority of which were in *gyrA* (D94 40/51; S91P 5/51; A90V 4/51). Other mutations in *gyrA* (1/51) and *gyrB* (3/51) were present in single isolates, but absent in a global resistance database^14^, suggesting that these may be novel fluoroquinolone resistance-conferring mutations. Two isolates had multiple resistance mutations in the *gyrA* gene (A94G-S91P and A90V-DA94G). The consistency between the drug susceptibility phenotypic results and *in-silico* predictions was variable for the two fluoroquinolones tested (moxifloxacin and ofloxacin). Whilst the sensitivities for both fluoroquinolones were 100%, the specificities were dissimilar (ofloxacin 85.7%; moxifloxacin 40.8%), which may be explained by differences in the critical concentration used for the drug susceptibility testing (moxifloxacin 2.5 μg/ml, ofloxacin 2.0 μg/ml).

Resistance across the aminoglycoside injectable drugs was associated with the *rrs* A1401G, which was linked with amikacin (3/3), capreomycin (3/3) and kanamycin (3/5) resistance. Kanamycin resistance was also observed with a mutation in the *eis* locus (−14C > T, 2/5). This mutation has been found to be related to low levels of kanamycin resistance^9^ but not associated with resistance to other aminoglycosides. A number of discrepancies were observed between the phenotypic test results and *in-silico* predictions for resistance. Three isolates had a resistant drug susceptibility test result for amikacin but did not have any known resistance mutations. Of these, one isolate did not have any mutations in known resistance genes, but the other two isolates had a (878 g > a) mutation in the *rrs* gene. This mutation has previously been reported to confer resistance to capreomycin^31^, and therefore potentially amikacin resistance too. Two isolates had resistant drug susceptibility test results for kanamycin but did not have any mutations in known resistance genes.

The presence of fluoroquinolone resistance mutations (n = 51; 63.0%) was overwhelmingly more common than aminoglycoside (amikacin/capreomycin) resistance mutations (n = 3; 3.7%), which is in contrast to other settings where resistance to second-line injectables was more common^32^. Inspection of the *in-silico* predictions revealed a significant proportion of isolates with fluoroquinolone resistance mutations to be pre-MDR-TB (n = 9; 17.6%). In particular, five isolates were resistant to fluoroquinolones and rifampicin but not isoniazid, one was resistant to fluoroquinolones and isoniazid but not rifampicin, and three were resistant to fluoroquinolones but sensitive to both rifampicin and isoniazid.

### Mutations in efflux pumps

Across the 81 isolates, we characterized fifty-five mutations in thirteen efflux pump genes (*Rv0194, Rv1217, Rv1218, drrA, drrB, Rv1258, Rv1634, Rv2688, Rv1273, Rv1819, Rv1458, Rv1877* and *Rv1250*) (Supplementary Table 3**)**. These mutations included twelve identified in previous work in Pakistan^12^. Mutation variants were observed in all thirteen efflux pump genes, with both SNPs and indels present (Supplementary Table 3**)**. Mutations in efflux genes were present in both susceptible and drug-resistant isolates, demonstrating that their potential role in resistance may be complex, and mechanisms may involve transcriptional or epigenetic effects that we did not consider.

### Evidence of transmission

We combined our study WGS data (n = 81) with those from a published Karachi study^19^ (n = 42). Potential transmission clusters were found by calculating the pairwise SNP distance between the 123 Pakistan isolates and using an established cut-off of <10 mutation differences^18^ (Supplementary Fig. 2). Eight clusters were found, with a maximum size of three isolates. Of these eight clusters, four contained only MDR-TB strains, three only XDR-TB strains, and one both MDR-TB and XDR-TB strains. Five clusters belonged to lineage 3, two to lineage 4, and one to lineage 1. Three of the clusters (the two lineage 4 clusters and a lineage 3 cluster) involved isolates from Khyber Pakhtunkhwa province. Overall, these data suggest ongoing transmission of MDR-TB and XDR-TB in Pakistan.

## Discussion

WGS is being used increasingly as a tool to assist epidemiological investigations and clinical and control program decision making in infectious diseases. However, most applications of WGS take place in developed countries, where the bacterial disease burden tends to be lower. Our study is the largest WGS analysis of drug resistant *M. tuberculosis* isolates from a high-burden TB region in Pakistan. In particular, the isolates were collected in Khyber Pakhtunkhwa province in North West Pakistan, a region that has been affected by recent armed conflict, social upheaval and refugee migration, and where operating an effective public health surveillance program has been difficult. CAS/Delhi was the most predominant strain-type identified in our analysis, and this is consistent with previous reports of lineage 3 strains dominating in South Asian populations^12,15,33,34^. The Beijing strain-type (lineage 2) was present in our study (11%), and although absent from previous WGS studies in Pakistan^12^, studies using spoligotyping have observed this strain-type in Pakistan before (e.g. 6% in^33^). It is unclear if the differences in the Beijing frequencies are due to the effects of increased prevalence, isolate selection, or geographical region. In general, Beijing strains are highly virulent and mobile, and their presence in Pakistan (and likely Afghanistan) is a concern for public health surveillance. Interestingly, in pre-MDR-TB isolates, the presence of rifampicin resistance mutations was more common than isoniazid resistance conferring mutations. This observation potentially indicates that resistance to rifampicin arises before isoniazid in this region, making this setting unique^35^. The high levels of fluoroquinolone (particularly ofloxacin) resistance in Pakistan have been observed by others^2^. This resistance, in some cases without evidence of rifampicin or isoniazid resistance, is worrisome and may be due to extensive and unregulated use of fluoroquinolones^2^.

The most frequent drug resistance conferring mutations identified in this study were already known, including in *katG* (*e.g*. S315T) and *Rv1482c*–*fabG1* intergenic region for isoniazid, *rpoB* (*e.g*. S450L and others in the RRDR) for rifampicin, *embB* (*e.g*. M306) for ethambutol, *gyrA* (*e.g*. D94) for fluoroquinolones, *rrs* (*e.g*. 1401a > g) for aminoglycosides, and *rpsL, rrs* and *giB* for streptomycin. The similarity of mutations observed and their frequency with previous Pakistan XDR-TB WGS data^19^ and global collections^9,14^ suggests that extensions of line probe assays and genotyping arrays to account for these mutations may be useful for disease control. However, we did identify novel potential resistance conferring mutations, including polymorphisms in *katG* (isoniazid), *ethA* (ethionamide), *gyrA* and *gyrA* (fluoroquinolones), and *pncA* (pyrazinamide). These mutations should be investigated and validated experimentally to determine their impact on drug minimum inhibitory concentrations and regimen efficacy. Our analysis has also revealed the potential transmission of drug resistant *M. tuberculosis* in Pakistan. However, a larger sample size and denser sampling frame will be required to fully characterize the degree of XDR-/MDR-TB transmission alongside genetic and non-genetic risk factors.

Overall, our work reveals the utility of WGS for the prediction of antimicrobial drug resistance, epidemiology and control activities in the Pakistan setting. The WGS data generated will serve as a baseline reference for future TB clinical, surveillance and control activities in Pakistan and the wider region.

## Conclusion

The application of WGS for TB clinical management and disease control will have the greatest benefit in complex community outbreaks in endemic regions, where epidemiological data availability may be sparse. Our study in the Khyber Pakhtunkhwa province in Pakistan has provided a baseline characterization of circulating known and putative drug resistance mutations, and identified potential MDR-TB transmission chains. These insights will assist future proactive TB patient management, and the deployment of anti-TB drug regimens and surveillance activities.

## Supplementary information

Supplementary tables and figures

## Data Availability

The accession codes for the raw sequence data are available in Supplementary Table 2.
