# Supplementary material for: Whole genome sequencing of drug resistant Mycobacterium tuberculosis isolates from a high burden tuberculosis region of North West Pakistan
Source: Sci Rep. 2019 Oct 18;9:14996. doi: 10.1038/s41598-019-51562-6 (PMC6802378; doi:10.1038/s41598-019-51562-6)
Supplement: Supplementary file 1 — Supplementary tables and figures [file 41598_2019_51562_MOESM1_ESM.pdf]

Whole genome sequencing of drug resistant *Mycobacterium tuberculosis* isolates from a high burden tuberculosis region of North West Pakistan

Abdul Jabbar [1,2,*], Jody E. Phelan [3], Paola Florez de Sessions[4], Taj Ali Khan[2], Hazir Rahman[5], Sadiq Noor Khan[1], Daire M. Cantillon[6], Leticia Muraro Wildner[6], Sajid Ali[7], Susana Campino [3], Simon J. Waddell [6,**], Taane G. Clark [3,8,*,**]

1 Department of Medical Lab Technology, University of Haripur, Haripur, Pakistan

2 Department of Microbiology, Kohat University of Science and Technology, Kohat, Pakistan

3 Faculty of Infectious and Tropical Diseases, London School of Hygiene and Tropical Medicine, Keppel Street, London WC1E 7HT, UK

4 Genome Institute of Singapore, 60 Biopolis St, Biopolis, Singapore

5. Department of Microbiology, Abdul Wali Khan University, Mardan, Pakistan

6 Department of Global Health and Infection, Brighton and Sussex Medical School, University of Sussex, Brighton, BN1 9PX, UK

7 Provincial TB Reference Lab, Hayatabad Medical Complex Peshawar, Pakistan

8 Faculty of Epidemiology and Population Health, London School of Hygiene and Tropical Medicine, Keppel Street, London WC1E 7HT, UK

** joint authors

*Corresponding authors

Abdul Jabbar

Department of Medical Lab Technology, University of Haripur, Haripur, Pakistan

Email: abdul.jabbar@uoh.gov.pk

Professor Taane Clark

Department of Infection Biology, London School of Hygiene & Tropical Medicine, London, UK

Taane.clark@lshtm.ac.uk

Supplementary table 1

Study data

ENA Accession	Region	Lineage	Drug resistance
ERR3335723	Dera Ismail Khan	3	MDR
ERR3335724	Peshawar	3	XDR
ERR3335725	Mardan	3	Drug-resistant
ERR3335726	Peshawar	3	MDR
ERR3335727	Dera Ismail Khan	3	MDR
ERR3335728	Peshawar	3	MDR
ERR3335729	Peshawar	3	MDR
ERR3335730	Peshawar	1.2.2	Drug-resistant
ERR3335731	Abbottabad	3	MDR
ERR3335732	Dera Ismail Khan	3.1.2	MDR
ERR3335733	Peshawar	4.5	MDR
ERR3335734	Swat	4	MDR
ERR3335735	Dera Ismail Khan	3	MDR
ERR3335736	Peshawar	4.5	MDR
ERR3335737	Abbottabad	3	MDR
ERR3335738	Peshawar	2.2.1	MDR
ERR3335739	Peshawar	3	XDR
ERR3335740	Peshawar	3	MDR
ERR3335741	Dera Ismail Khan	3	MDR
ERR3335742	Dera Ismail Khan	2.2.1	MDR
ERR3335743	Swat	3	XDR
ERR3335744	Peshawar	4.5	MDR
ERR3335745	Peshawar	4.5	MDR
ERR3335746	Peshawar	1.2.2	Drug-resistant
ERR3335747	Swat	4	MDR
ERR3335748	Peshawar	3	MDR
ERR3335749	Dera Ismail Khan	2.2.1	MDR
ERR3335750	Abbottabad	3	MDR
ERR3335751	Dera Ismail Khan	2.2.1	MDR
ERR3335752	Dera Ismail Khan	3	MDR
ERR3335753	Dera Ismail Khan	1.1.2	Drug-resistant
ERR3335754	Peshawar	3	MDR
ERR3335755	Peshawar	3	Drug-resistant
ERR3335756	Peshawar	3	MDR
ERR3335757	Peshawar	4.5	MDR
ERR3335758	Abbottabad	3	MDR
ERR3335759	Peshawar	3	MDR
ERR3335760	Swat	3	MDR
ERR3335761	Dera Ismail Khan	3	MDR
ERR3335762	Dera Ismail Khan	3	MDR
ERR3335763	Peshawar	2.2.1	MDR
ERR3335764	Swat	3	MDR
ERR3335765	Abbottabad	3	Drug-resistant
ERR3335766	Peshawar	3	MDR
ERR3335767	Abbottabad	3	MDR
ERR3335768	Peshawar	4.5	MDR
ERR3335769	Peshawar	3	Drug-resistant
ERR3335770	Abbottabad	3	MDR

ERR3335771	Peshawar	4.5	MDR
ERR3335772	Peshawar	3	MDR
ERR3335773	Abbottabad	3	MDR
ERR3335774	Abbottabad	3	MDR
ERR3335775	Abbottabad	4.9	MDR
ERR3335776	Peshawar	3	XDR
ERR3335777	Peshawar	3	MDR
ERR3335778	Dera Ismail Khan	3	Drug-resistant
ERR3335779	Peshawar	3.1.2.1	MDR
ERR3335780	Peshawar	3	Drug-resistant
ERR3335781	Peshawar	3	MDR
ERR3335782	Abbottabad	3	Drug-resistant
ERR3335783	Peshawar	3	Sensitive
ERR3335784	Peshawar	2.2.1	MDR
ERR3335785	Peshawar	2.2.1	MDR
ERR3335786	Dera Ismail Khan	3	Drug-resistant
ERR3335787	Dera Ismail Khan	3	Drug-resistant
ERR3335788	Peshawar	3	MDR
ERR3335789	Peshawar	3	MDR
ERR3335790	Abbottabad	3	MDR
ERR3335791	Peshawar	2.2.1	MDR
ERR3335792	Peshawar	3	MDR
ERR3335793	Peshawar	4.2.1	MDR
ERR3335794	Abbottabad	3	MDR
ERR3335795	Peshawar	3	MDR
ERR3335796	Dera Ismail Khan	3	Drug-resistant
ERR3335797	Dera Ismail Khan	3	MDR
ERR3335798	Peshawar	2.2.1	MDR
ERR3335799	Peshawar	3	MDR
ERR3335800	Peshawar	4.8	MDR
ERR3335801	Peshawar	3	XDR
ERR3335802	Peshawar	3	MDR
ERR3335803	Swat	3	MDR

MDR = multi-drug resistant TB, XDR = extensively drug resistant TB; Drug-resistant = non-MDR/XDR resistant as determined by *in-silico* prediction using TB Profiler software

Supplementary table 2

Frequency of drug resistance related mutations in candidate genes

Drug	Gene	Mutation	Study N	Study %	Global * N	Global * %
Amikacin	*rrs*	1401a>g	3/3	100	291/349	83.4
Capreomycin	*rrs*	1401a>g	3/3	100	307/404	76.0
Ethambutol	*embA*	-12C>T	3/61	4.9	115/2662	4.3
Ethambutol	*embA*	-16C>T	1/61	1.6	60/2662	2.3
Ethambutol	*embB*	Met306Ile	20/61	32.8	567/2662	21.3
Ethambutol	*embB*	Met306Val	15/61	24.6	840/2662	31.6
Ethambutol	*embB*	Met306Leu	10/61	16.4	34/2662	1.3
Ethambutol	*embB*	Gln497Arg	5/61	8.2	207/2662	7.8
Ethambutol	*embB*	Gly406Ala	4/61	6.6	318/2662	12.0
Ethambutol	*embB*	Gln497Lys	3/61	4.9	20/2662	0.8
Ethambutol	*embB*	Gly406Asp	3/61	4.9	75/2662	2.8
Ethambutol	*embB*	Gly406Ser	3/61	4.9	37/2662	1.4
Ethambutol	*embB*	Asp354Ala	1/61	1.6	146/2662	5.5
Ethambutol	*embB*	Gln497Pro	1/61	1.6	20/2662	0.8
Ethambutol	*embB*	Tyr319Ser	1/61	1.6	18/2662	0.7
Ethionamide	*ethA*	1054_1054del	1/15	6.7	2/347	0.6
Ethionamide	*ethA*	1200_1201del	1/15	6.7	0/347	0.0
Ethionamide	*ethA*	1216_1217ins	1/15	6.7	0/347	0.0
Ethionamide	*ethA*	140_140del	1/15	6.7	0/347	0.0
Ethionamide	*ethA*	314_315ins	1/15	6.7	0/347	0.0
Ethionamide	*ethA*	377_378insGGCTA	1/15	6.7	0/347	0.0
Ethionamide	*ethA*	599_599del	1/15	6.7	0/347	0.0
Ethionamide	*ethA*	269Q>269*	1/15	6.7	0/347	0.0
Ethionamide	*ethA*	347Q>347*	1/15	6.7	0/347	0.0
Ethionamide	*fabG1*	-15C>T	7/15	46.7	190/347	54.8
FQ	*gyrA*	Asp94Gly	26/51	51.0	153/527	29.0
FQ	*gyrA*	Asp94Ala	9/51	17.6	62/527	11.8
FQ	*gyrA*	Ser91Pro	5/51	9.8	40/527	7.6
FQ	*gyrA*	Ala90Val	4/51	7.8	152/527	28.8
FQ	*gyrA*	Asp94Asn	3/51	5.9	22/527	4.2
FQ	*gyrA*	Asp94Tyr	2/51	3.9	20/527	3.8
FQ	*gyrA*	Gly88Cys	1/51	2.0	0/527	0
FQ	*gyrB*	Glu501Asp	1/51	2.0	0/527	0
FQ	*gyrB*	Ser447Phe	1/51	2.0	0/527	0
FQ	*gyrB*	Thr500Asn	1/51	2.0	0/527	0
Isoniazid	*ahpC*	-54C>T	1/68	1.5	15/5423	0.3
Isoniazid	*fabG1*	-15C>T	7/68	10.3	877/5423	16.2
Isoniazid	*katG*	Ser315Thr	61/68	89.7	4071/5423	75.1
Isoniazid	*katG*	1328_1328del	1/68	1.5	0/5423	0.0
Isoniazid	*katG*	338_338del	1/68	1.5	0/5423	0.0
Isoniazid	*katG*	596_596del	1/68	1.5	0/5423	0.0
Isoniazid	*katG*	Ser315Asn	1/68	1.5	83/5423	1.5
Isoniazid	*katG*	Tyr155Cys	1/68	1.5	4/5423	0.1
Kanamycin	*eis*	-14C>T	2/5	40.0	17/669	2.5
Kanamycin	*rrs*	1401a>g	3/5	60.0	531/669	79.4
Pyrazinamide	*pncA*	-11A>G	4/43	9.3	77/1946	4.0
Pyrazinamide	*pncA*	Val180Phe	4/43	9.3	4/1946	0.2

Pyrazinamide	*pncA*	His71Arg	3/43	7.0	2/1946	0.1
Pyrazinamide	*pncA*	316_317insG	2/43	4.7	0/1946	0.0
Pyrazinamide	*pncA*	Ala146Val	2/43	4.7	10/1946	0.5
Pyrazinamide	*pncA*	Arg140Gly	2/43	4.7	0/1946	0.0
Pyrazinamide	*pncA*	His51Arg	2/43	4.7	13/1946	0.7
Pyrazinamide	*pncA*	Leu19Arg	2/43	4.7	0/1946	0.0
Pyrazinamide	*pncA*	Thr47Ser	2/43	4.7	0/1946	0.0
Pyrazinamide	*pncA*	Thr76Pro	2/43	4.7	16/1946	0.8
Pyrazinamide	*pncA*	194_203del	1/43	2.3	0/1946	0.0
Pyrazinamide	*pncA*	393_394insG	1/43	2.3	6/1946	0.3
Pyrazinamide	*pncA*	393_394insGG	1/43	2.3	26/1946	1.3
Pyrazinamide	*pncA*	426_427insGA	1/43	2.3	0/1946	0.0
Pyrazinamide	*pncA*	440_441insCG	1/43	2.3	0/1946	0.0
Pyrazinamide	*pncA*	2288682_2289086del	1/43	2.3	0/1946	0.0
Pyrazinamide	*pncA*	Asp12Ala	1/43	2.3	11/1946	0.6
Pyrazinamide	*pncA*	Asp49Asn	1/43	2.3	7/1946	0.4
Pyrazinamide	*pncA*	Gln10Pro	1/43	2.3	280/1946	14.4
Pyrazinamide	*pncA*	Gln141Pro	1/43	2.3	19/1946	1.0
Pyrazinamide	*pncA*	Gly78Ser	1/43	2.3	1/1946	0.1
Pyrazinamide	*pncA*	His57Pro	1/43	2.3	2/1946	0.1
Pyrazinamide	*pncA*	His57Tyr	1/43	2.3	2/1946	0.1
Pyrazinamide	*pncA*	His71Tyr	1/43	2.3	22/1946	1.1
Pyrazinamide	*pncA*	Ile133Thr	1/43	2.3	24/1946	1.2
Pyrazinamide	*pncA*	Lys96Thr	1/43	2.3	10/1946	0.5
Pyrazinamide	*pncA*	Met175Thr	1/43	2.3	2/1946	0.1
Pyrazinamide	*pncA*	Pro54Leu	1/43	2.3	26/1946	1.3
Pyrazinamide	*pncA*	Ser164Pro	1/43	2.3	4/1946	0.2
Pyrazinamide	*pncA*	Thr142Ala	1/43	2.3	4/1946	0.2
Pyrazinamide	*pncA*	Thr76Ile	1/43	2.3	1/1946	0.1
Pyrazinamide	*pncA*	Val139Ala	1/43	2.3	11/1946	0.6
Rifampicin	*rpoB*	Ser450Leu	56/76	73.7	2975/4618	64.4
Rifampicin	*rpoB*	Asp435Val	4/76	5.3	296/4618	6.4
Rifampicin	*rpoB*	His445Tyr	3/76	3.9	230/4618	5.0
Rifampicin	*rpoB*	Asp435Tyr	2/76	2.6	104/4618	2.3
Rifampicin	*rpoB*	His445Asp	2/76	2.6	162/4618	3.5
Rifampicin	*rpoB*	Leu430Pro	2/76	2.6	55/4618	1.2
Rifampicin	*rpoB*	Leu452Pro	2/76	2.6	142/4618	3.1
Rifampicin	*rpoB*	Ser450Phe	2/76	2.6	22/4618	0.5
Rifampicin	*rpoB*	1306_1308del	1/76	1.3	2/4618	0.0
Rifampicin	*rpoB*	Asp435Gly	1/76	1.3	71/4618	1.5
Rifampicin	*rpoB*	His445Arg	1/76	1.3	45/4618	1.0
Rifampicin	*rpoB*	His445Leu	1/76	1.3	56/4618	1.2
Rifampicin	*rpoB*	His445Pro	1/76	1.3	4/4618	0.1
Rifampicin	*rpoB*	Leu430Arg	1/76	1.3	12/4618	0.3
Rifampicin	*rpoB*	Ser450Trp	1/76	1.3	83/4618	1.8
Rifampicin	*rpoC*	Ile491Thr	7/76	9.2	91/4618	2.0
Rifampicin	*rpoC*	Ile885Val	1/76	1.3	8/4618	0.2
Streptomycin	*gid*	102_102del	6/42	14.3	12/1408	0.9
Streptomycin	*gid*	Ala80Pro	2/42	4.8	12/1408	0.9
Streptomycin	*rpsL*	Lys43Arg	12/42	28.6	649/1408	46.1
Streptomycin	*rpsL*	Lys88Arg	3/42	7.1	104/1408	7.4
Streptomycin	*rpsL*	Lys88Met	1/42	2.4	3/1408	0.2
Streptomycin	*rrs*	514a>c	12/42	28.6	128/1408	9.1

Streptomycin	*rrs*	517c>t	3/42	7.1	57/1408	4.1
Streptomycin	*rrs*	462c>t	1/42	2.4	2/1408	0.1
Streptomycin	*rrs*	905c>g	1/42	2.4	0/1408	0.0
Streptomycin	*rrs*	906a>g	1/42	2.4	6/1408	0.4

*** from [14]; FQ = Fluoroquinolones, N = number of isolates determined as resistant in the respective studies**

Supplementary table 3

Mutations in efflux pump genes in the Pakistan samples and a global drug resistance dataset*

Gene	Mutation	Type	Our Frequency (n=81)	Global Pan Susc. (n=5,949)	Global Drug-res. (n=2,002)	Global MDR (n=3,886)	Global XDR (n=354)
Rv0194	**Met74Thr**	missense	80	4582	1573	2533	269
Rv2688c	**Pro156Thr**	missense	79	4095	1482	2403	264
Rv1458c	-102G>A	Non-coding	77	488	52	116	8
Rv1218c	**Gln243Arg**	missense	70	3297	1106	1907	210
Rv1634	Gly198Arg	missense	69	2575	799	1371	174
Rv1273c	**Gly462Lys**	missense	52	1023	155	153	27
Rv0194	Pro1098Leu	missense	9	710	397	1090	140
Rv1217c	Ala173Thr	missense	9	706	392	1056	145
Rv1458c	Thr133Ala	missense	9	605	324	968	144
Rv1258c	580_581insC	frameshift	7	684	352	1000	84
Rv1258c	Pro414Ser	missense	6	23	3	2	0
Rv1250	Pro470Arg	missense	5	13	4	4	0
bacA	**Ile603Val**	missense	3	766	238	110	5
drrA	**His309Asp**	missense	3	765	237	111	5
Rv0194	-202G>A	Non-coding	3	727	171	111	5
Rv1250	**Arg278Gly**	missense	3	766	237	110	5
Rv1458c	Ala4Gly	missense	3	250	49	52	0
Rv1634	Ala394Val	missense	3	0	0	0	0
Rv1634	Val438Met	missense	3	0	0	0	0
Rv0194	Gly1057Ala	missense	2	13	0	2	0
Rv0194	**Gly170Val**	missense	2	102	48	40	2
Rv1273c	**Ser118Gly**	missense	2	77	44	31	2
Rv1877	**Ile534Thr**	missense	2	100	47	40	2
bacA	1650_1651insG	frameshift	1	0	1	7	0
bacA	Asp546Ala	missense	1	3	3	1	0
bacA	Ile273Thr	missense	1	140	50	94	2
drrA	Ala24Thr	missense	1	7	5	6	0
drrB	Ala264Val	missense	1	0	0	0	0
Rv0194	-122G>C	Non-coding	1	76	8	14	3
Rv0194	2029_2030insC	frameshift	1	0	0	0	0
Rv0194	-292A>G	Non-coding	1	0	0	0	0
Rv0194	971_972del	frameshift	1	9	1	1	0
Rv0194	228671_230461del	Large deletion	1	0	0	0	0
Rv0194	230425_230461del	Large deletion	1	0	0	0	0
Rv0194	Ala596Thr	missense	1	0	0	0	0
Rv0194	Arg954Gln	missense	1	0	0	0	0
Rv0194	Gln1072Arg	missense	1	24	6	4	0
Rv0194	Gln188Arg	missense	1	6	4	6	0

Rv0194	Gln818Arg	missense	1	11	1	0	0
Rv0194	His16Leu	missense	1	2	0	1	0
Rv0194	His448Tyr	missense	1	1	0	0	0
Rv0194	Ser421Phe	missense	1	2	0	1	0
Rv0194	Val127Gly	missense	1	0	0	0	0
Rv1217c	Gly294Arg	missense	1	0	0	0	0
Rv1217c	Gly437Cys	missense	1	0	0	0	0
Rv1217c	**Leu151Arg**	missense	1	131	35	16	1
Rv1218c	Gly71Asp	missense	1	2	1	0	2
Rv1258c	580_581insC	frameshift	1	0	0	0	0
Rv1258c	581_582insA	frameshift	1	0	0	0	0
Rv1273c	Glu444Asp	missense	1	0	0	0	0
Rv1634	**Ile47Val**	missense	1	454	89	49	3
Rv1877	Pro168Ser	missense	1	9	1	2	0
Rv1877	Val593Ala	missense	1	0	0	0	0
Rv2688c	-139A>G	Non-coding	1	436	63	89	1
Rv2688c	Ala209Asp	missense	1	3	0	0	0

* from reference [14]; bolded mutations are those present in a previous Pakistan study[12]; Pan Susc. = pan susceptibility; Drug-res. = non-MDR/XDR resistant; MDR = multi-drug resistant TB, XDR = extensively drug resistant TB

Supplementary figure 1

Geographical map showing the source of the *Mycobacterium tuberculosis* samples (n=81)

Regions are colored according to the number of samples contributed. Pie charts show the proportion of drug resistance types from each region.

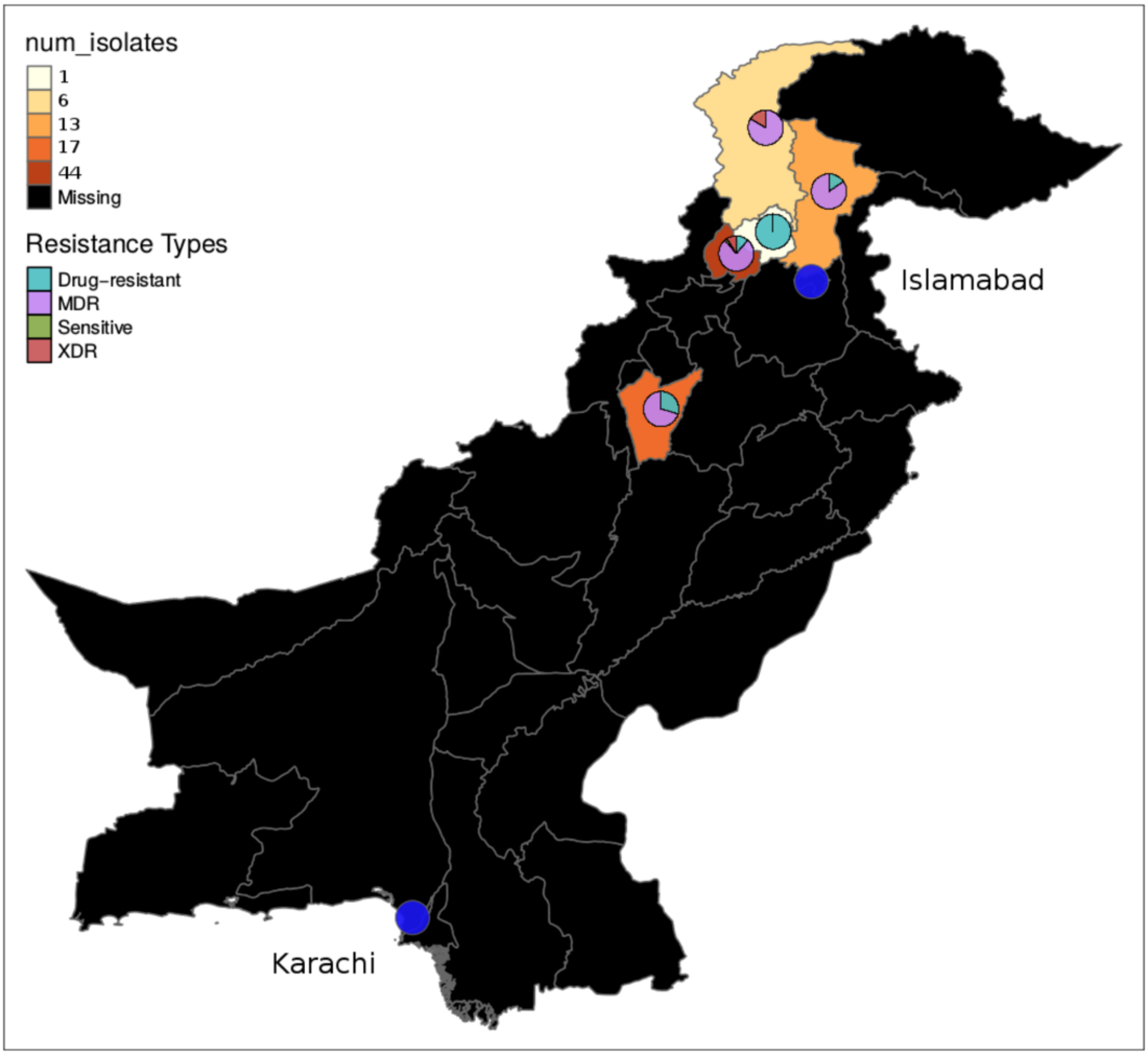

MDR = multi-drug resistant TB, XDR = extensively drug resistant TB; Drug-resistant = non-MDR/XDR resistant

Supplementary figure 2

Transmission analysis across all Pakistan samples (n=123)

(a) Circular phylogenetic tree of Pakistan isolates (81 this study; 42 from Karachi study[19]) show evidence of eight potential transmission clusters (<10 SNPs difference), highlighted by red boxes

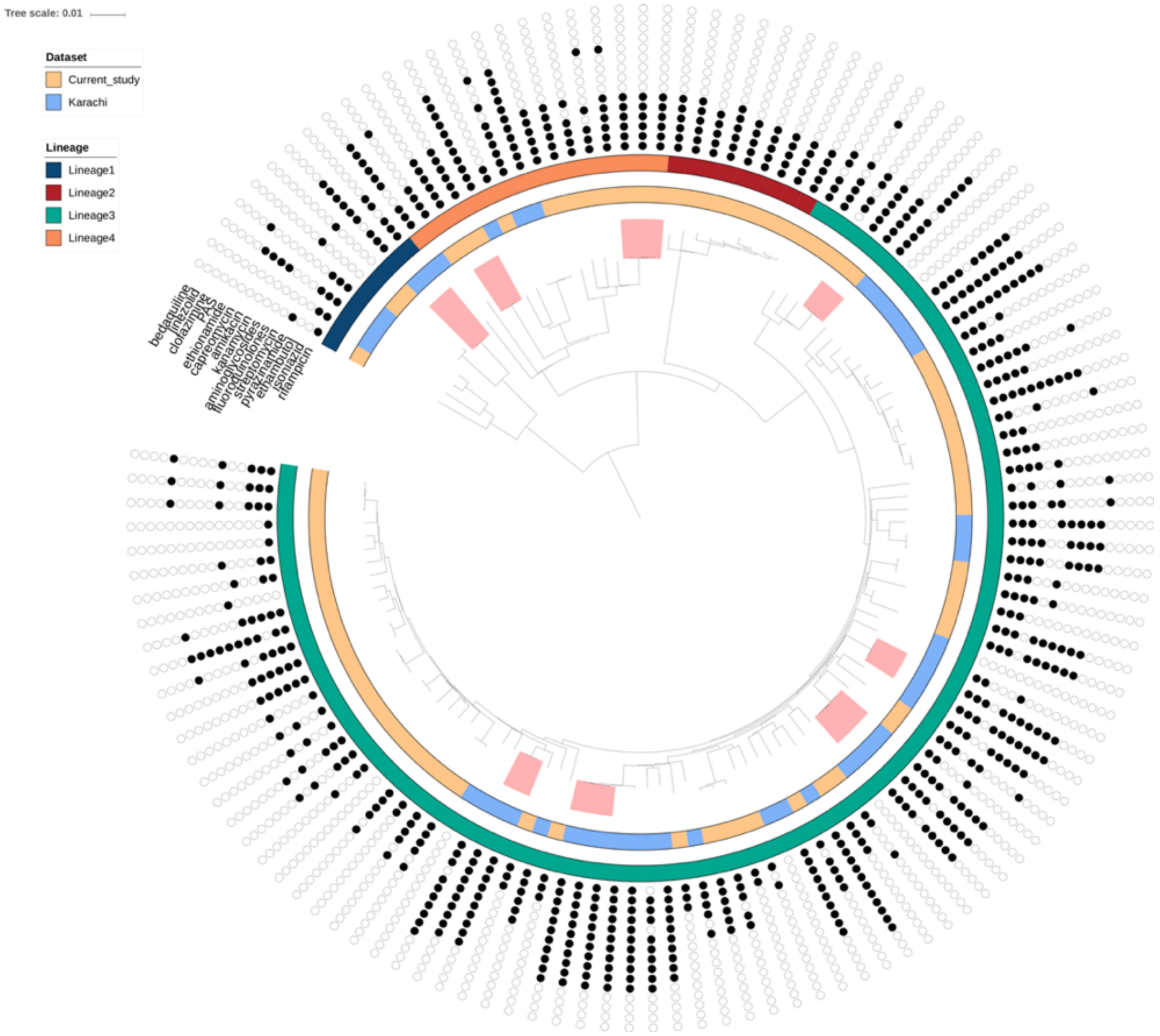

(b) Eight putative MDR/XDR-TB transmission chains (isolates with <10 SNPs difference) with three from our study (2 lineage 4, one lineage 3), and five from the Karachi study[19]

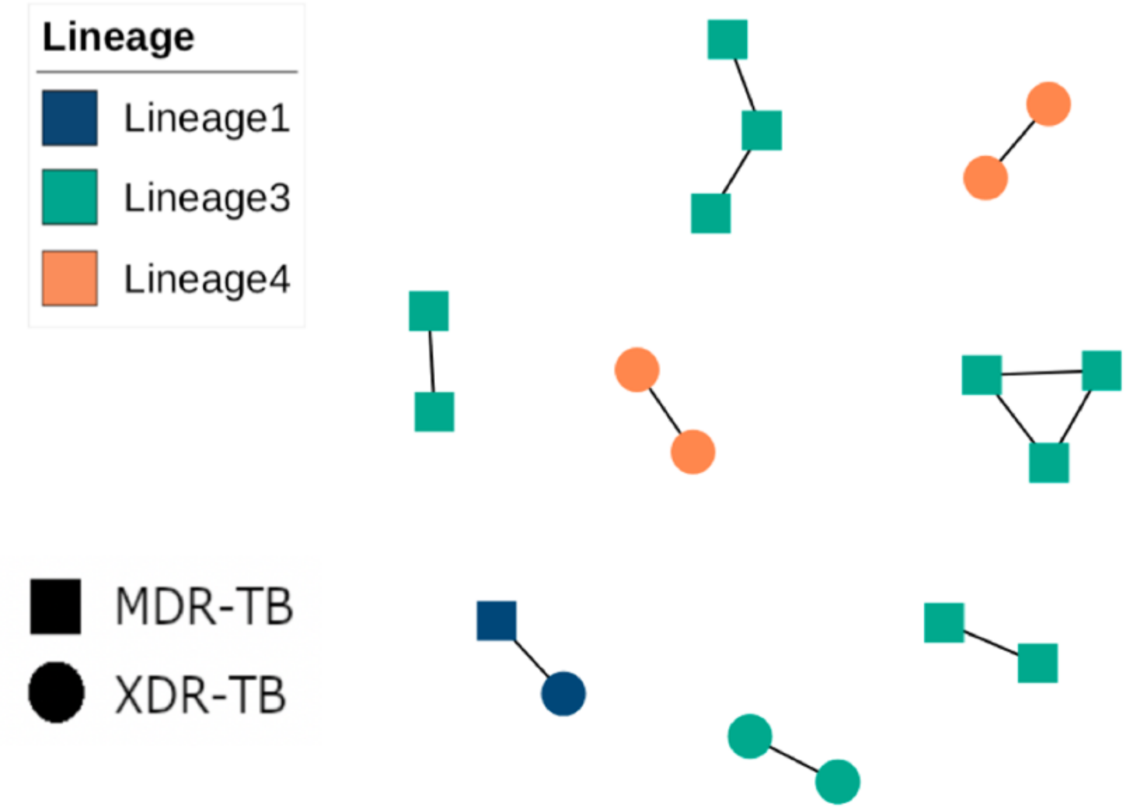

Supplementary figure 3

Mutations in the *pncA* gene

The top panel displays the frequencies of the mutations found in the Pakistan dataset (n=81), with their corresponding frequencies in a global dataset*. The middle panel displays the depth of coverage in the ERR3335748 isolate for which a large 405bp deletion was detected.

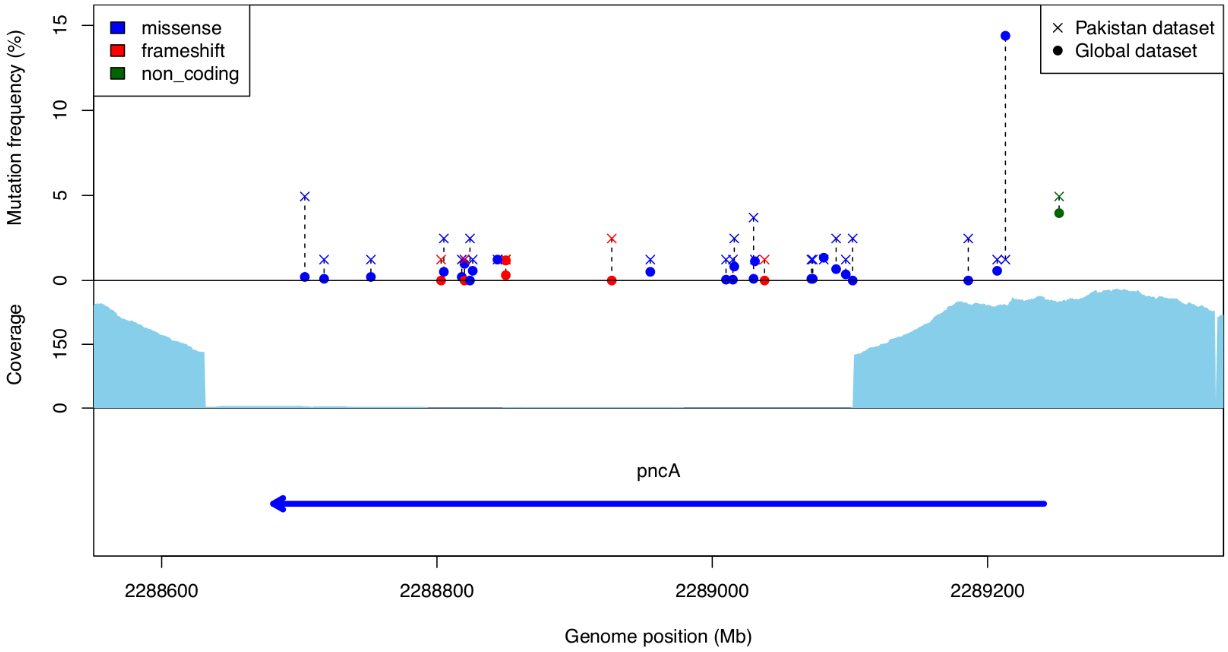

*** reference** [14]
